# Atorvastatin Inhibits Ferroptosis of H9C2 Cells by regulatingSMAD7/Hepcidin Expression to Improve Ischemia-Reperfusion Injury

**DOI:** 10.1155/2022/3972829

**Published:** 2022-11-08

**Authors:** You Peng, Bin Liao, Yan Zhou, Wei Zeng, Zhi-Yu Zeng

**Affiliations:** ^1^Department of Geriatric Cardiology & Guangxi Key Laboratory of Precision Medicine in Cardio-Cerebrovascular Diseases Control and Prevention & Guangxi Clinical Research Center for Cardio-Cerebrovascular Diseases, The First Affiliated Hospital of Guangxi Medical University, Nanning, Guangxi, China; ^2^Department of Geriatric, Hunan Provincial People's Hospital, The First Affiliated Hospital of Hunan Normal University, Changsha, Hunan, China; ^3^Hunan Research Institute of Geriatrics, Changsha, Hunan, China

## Abstract

**Background:**

Ferroptosis plays a key role in cardiomyopathy. Atorvastatin (ATV) has a protective effect on ischemia-reperfusion (I/R) cardiomyopathy. The purpose of this study is to elucidate the mechanism of ATV in I/R injury.

**Methods:**

H9C2 cells and cardiomyopathy rats were induced by hypoxia/reoxygenation (H/R) and I/R to construct *in vitro* and *in vivo* models. Cell viability was determined by CCK8. Cardiac histopathology was observed by HE staining. Transmission electron microscope (TEM) was used to observe the mitochondrial morphology. The reactive oxygen species (ROS) content in cells was analyzed by the biochemical method. ELISA was conducted to calculate the concentrations of total iron/Fe^2+^ and hepcidin. The expression of ferroptosis and SMAD pathway-related genes were detected by qPCR. Western blot was performed to detect the expression levels of ferroptosis and SMAD pathway-related proteins.

**Results:**

In H9C2 cells, ATV reversed the decline in cell viability, mitochondrial shrinkage, and ROS elevation induced by erastin or H/R. The concentration of total iron and Fe^2+^ in H/R-induced H9C2 cells increased, and the protein expression of FPN1 decreased. After ATV treatment, the concentration of total iron and Fe^2+^ decreased, and the protein expression of FPN1 increased. The expression of the SMAD7 gene in H/R-induced H9C2 cells decreased, and the expression of the hepcidin gene increased, which were reversed by ATV. When SMAD7 was knocked down, ATV treatment failed to produce the above effect. ATV also improved ferroptosis in I/R rat myocardium through the SMAD7/hepcidin pathway.

**Conclusions:**

ATV reversed the decline in H9C2 cell viability, mitochondrial shrinkage, and ROS elevation, and improved the myocardium ferroptosis through the SMAD7/hepcidin pathway in I/R rat.

## 1. Introduction

In recent years, programmed myocardial cell necrosis induced by iron-overload has been found to promote the occurrence of cardiomyopathy [1]. This death does not respond to apoptosis, necrosis, or autophagy inhibitors, but is effectively inhibited by iron-chelating agents, hence, it is named iron-dependent cell death (ferroptosis) [2]. Ferroptosis is characterized by excessive lipid peroxidation of polyunsaturated fatty acids (PUFAs) in the biofilms, resulting in cell membrane perforation and cell death [3]. Xuexian Fang et al. found that ferroptosis played a key role in doxorubicin-induced cardiomyopathy [4]. Targeting ferroptosis for the prevention and treatment of cardiomyopathy has heralded new prospects.

Atorvastatin (ATV) is a statin that inhibits the enzyme hydroxymethylglutaryl-CoA reductase and works by down-regulating the LDL cholesterol levels [5]. ATV has anti-inflammatory, antioxidant, fatty acid metabolism and other functions [6, 7]. In clinical practice, ATV was widely used in the treatment of hyperlipidemia, coronary artery disease, and other atherosclerotic diseases [8]. In addition, the protective effect of ATV in I/R cardiomyopathy and diabetic cardiomyopathy has been repeatedly confirmed by several studies [9, 10]. More and more studies have confirmed the regulatory effect of ATV on ferroptosis. However, studies on how ATV affects ferroptosis in cardiomyopathy were incomplete.

Bone Morphogenetic Protein (BMP)-small mothers against decapentaplegic (SMAD) signaling pathway was the core of regulating hepcidin transcription [11]. SMAD7 was an inhibitory SMAD protein that mediated the negative feedback loop of BMP signal transduction to effectively inhibit the expression of hepcidin [12]. Previous studies have found that the BMP-SMAD signal axis in hepatocytes was the core signal mediating the expression and secretion of hepcidin induced by the IL-6 signal [13]. Hepcidin is a hormone that regulates the absorption, storage, and transport of iron throughout the body [14]. Hepcidin deficiency disrupts iron homeostasis leading to the common iron-overload syndrome [15]. These studies suggested that the effect of ATV on the ferroptosis of cardiomyopathy might be realized by regulating SMAD7/hepcidin.

We hypothesized that ATV might inhibit ferroptosis of H9C2 cells by regulating SMAD7/hepcidin expression to improve I/R injury. This study aims to explore the mechanism of ATV in ischemia-reperfusion (I/R) injury through *in vitro* and *in vivo* models, which provide a new treatment strategy for patients with iron-overload cardiomyopathy.

## 2. Materials and Methods

### 2.1. Cell Culture and Treatment

Rat cardiomyocytes H9C2 (CBP60588, Cobioer) cells were maintained in a DMEM medium (D5796,Sigma) supplemented with 10% FBS (10099141, Gibco) and multiplied at 37°C at 5% CO_2_. Penicillin and streptomycin (SV30010, Beyotime) were added to prevent bacterial contamination. The cells were randomly divided into six groups: control, erastin, H/R, ATV, erastin + ATV, and H/*R* + ATV groups. The cells were treated differently according to groups. The cells erastin and erastin + ATV groups were treated with erastin (5 *μ*M) for 24 h [16]. The steps of cell hypoxia/reoxygenation (H/R) stimulation were as follows: H9C2 cells were exposed to hypoxia (1% O_2_/94% N_2_/5% CO_2_) for 4 h, followed by oxygen for 2 h [17]. The working concentration of ATV was 1 *μ*M, and the incubation time was 24 h [18].

In order to study the mechanism of ATV, the cells were divided into four groups: H/R, H/*R* + SMAD7i, H/*R* + ATV, and H/*R* + SMAD7i + ATV groups. SMAD7i were constructed by transfecting siRNA targeting SMAD7 into H9C2 cells. siRNA targeting rat SMAD7 (5′-AGGCAUUCCUCGGAAGUCATT-3′) and the negative control (NC) were synthetic by Tsingke Biotechnology Co., Ltd. [19]. Lipofectamine 2000 (12566014, ThermoFisher) reagent diluted with a serum-free medium was used to aid transfection. DMEM containing FBS was replaced 4 h after transfection.

### 2.2. Animal Experiment and Grouping

A total of 18 Wistar rats (250∼300 g) were purchased from Hunan slake Jingda experimental animal Co., Ltd. The rats were randomly divided into the Sham group, I/R group, and I/*R* + ATV group (*n* = 6/group).They received standard diet and water before myocardial I/R. Rats in the I/*R* + ATV group were orally treated with ATV (10 mg/kg/d) for 2 weeks before myocardial I/R (9).The Sham and I/R model rats [9, 20] were constructed as follows: The rats were anesthetized with sodium pentobarbital (50 mg/kg, intraperitoneal injection), ligation of the left anterior descending branch with 4-0 silk thread for 30 min, and then reperfusion for 180 min. In the sham control group, the entire procedure was performed with silk thread passing below the coronary artery, but the LAD coronary artery was not ligated. At the end of reperfusion, the rats were given excessive isoflurane for 10 min and sacrificed by bloodletting. Then the rat myocardial tissues were isolated for subsequent detection.

### 2.3. Cell Count Kit-8 assay (CCK8)

The cells were decomposed into single cells by trypsin digestion solution (C0201, Beyotime) and inoculated into a 96-well plate (0030730119, Eppendorf) at a density of 5 × 10^3^ cells per well. Each group was set with 3 wells. A 100 *μ*L complete culture medium containing CCK8(NU679, Dojindo) was added to each well. The cells were incubated at 37°C for 4 h at 5% CO_2_ [21]. Then the optical density (OD) value at 450 nm was analyzed by an abio-tek microplate (MB-530, HEALES).

### 2.4. HE Staning

Rat myocardium was immobilized in 4% paraformaldehyde for 24 h and then dehydrated in a gradient of 20% and 30% sucrose solution. The myocardial tissues of rats were embedded in paraffin, sequentially sliced, connected to the treated slides, and baked at 60°C for 12 h. The sections were dewaxed to water by xylene and gradient ethanol (75∼100%).Hematoxylin and eosin (Wellbio) were used for section staining. The sections were observed with a microscope (BA210T, Motic).

### 2.5. Transmission Electron Microscope (TEM)

The cells were fixed with 2.5% glutaraldehyde and 1% osmium. Gradient concentrations of ethanol were used to dehydrate cells. The cells were incubated with 1 : 1 epoxy propane-epoxy resin and pure epoxy resin for 2 h, respectively. The cells were coated with pure epoxy resin and baked at 40°C for 12 h, followed by 60°C for 48 h. The cells were stained with lead and uranium salts and observed by transmission electron microscopy (JEM1400, Japan Electronics). Images were recorded with a Morada G3 digital camera.

### 2.6. Reactive Oxygen Species (ROS) Detection

ROS kits (S0033S, Beyotime) were used to detect the ROS level in cells. The mother solution of DCFH-DA was diluted with a serum-free medium to obtain 10 *μ*M of DCFH-DA working solution. Then the culture medium was removed. The cells were immersed with DCFH-DA working solution. The cells were placed in a 37°C cell culture chamber and incubated for 20 min. The cells were washed three times with a serum-free cell culture medium to fully remove DCFH-DA that did not enter the cells. The cells were digested into a single-cell suspension using trypsin digestion solution. The fluorescence signal was detected by flow cytometry.

In addition, we used C11-BODIPY fluorescent probe (D3861, thermofisher) to detect the ROS level again. In brief, the mother solution was obtained by adding 396 ul of DMSO to 1 mg of fluorescent probe at a concentration of 5 mM. The cells were diluted in the ratio of 1 : 1000 and stained at 37°C for 30 min in darkness, washed with PBS for three times in darkness, and then tested by flow cytometry.

### 2.7. Western Blot

Total protein was extracted with RIPA lysate (P0013 B, Beyotime) and quantified with a BCA protein quantification kit. SDS-PAGE electrophoresis was performed to separate proteins. The target proteins were then transferred to a cellulose nitrate (NC) membrane. The NC membrane was sealed with skim milk (5%). Then the membrane was incubated with ACSL4 (1 : 2000, 22401-1-AP, Proteintech), COX2 (1 : 1000, 66351-1-Ig, Proteintech), CPX4 (1 : 5000, 67763-1-Ig, Proteintech), SLC7A11 (1 : 2000, ab175186, Proteintech), SMAD7 (1 : 1000, 25840-1-AP, Proteintech), FPN1 (1 : 800, 26601-1-AP, Proteintech), p-SMAD1 (1 : 2000, 28865-1-AP, Proteintech), Hepcidin (1 : 3000, ab30760, Abcam), SMAD1 (1 : 800, 10429-1-AP, Proteintech), and GAPDH (1 : 5000,10494-1-AP, Proteintech). Then it was incubated with secondary antibodies HRP goat antimouse IgG (1 : 5000, AWS0001a, Abiowell) and HRP goat antirabbit IgG (1 : 6000, AWS0002a, Abiowell)for 90 min, respectively. The protein bands were visualized using SuperECL Plus luminescence solution (K-12045-D50, Advansta). Chemiscope6100 (CLINX) was used to capture images.

### 2.8. Enzyme-Linked Immunosorbent Assay (ELISA)

Rat Hepc ELISA Kit (E-EL-R0500c) was purchased from Elabscience Biotechnology Co., Ltd. Iron Assay kit (ab83366) was purchased from Abcam. All steps were carried out in strict accordance with the instructions. After the reaction was terminated, the OD value of each well at 450 nm was detected by a bio-tek microplate (MB-530, HEALES).

### 2.9. Quantitative Polymerase Chain Reaction (qPCR)

The trizol was used to extract total RNA from cells and rat myocardial tissues. Primers of target genes (Table 1) were synthesized by Tsingke Biotechnology Co., Ltd. The cDNA was obtained by reverse transcription of mRNA using HiFscript cDNA Synthesis Kit (CW2569, CWBIO). A fluorescent quantitative PCR instrument (PIKOREAL96, Thermo) was used to amplify cDNA and detect the fluorescence signal. GAPDH was used as an internal reference. The relative expression levels of target genes were calculated by the 2^−ΔΔCt^ method.

### 2.10. Statistical Analysis

GraphPad Prism (8.0.1) was used to analyze data. Data were presented as the mean ± standard deviation. Unpaired students' *t*-test was used to compare two groups; one-way analysis of variance (ANOVA) followed by Tukey post hoc test was utilized for multiple groups. A statistical significance was defined at *P* < 0.05.

## 3. Results

### 3.1. ATV Inhibited the Ferroptosis of H9C2 Cells

**Figure 1:**
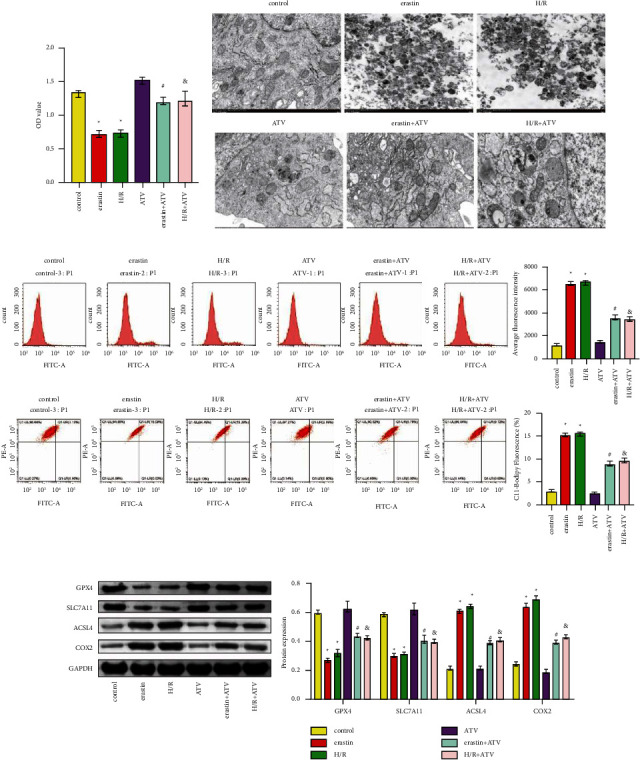
ATV inhibited the ferroptosis of H9C2 cells. (a). CCK8 was performed to measure cell vitality. (b). TEM was conducted to observe mitochondrial morphology. Magnification = 10 k and Scale bar = 1.0 *μ*m. (c). ROS level was detected by DCFH-DA solution and C11-BODIPY fluorescent probe in H9C2 cells with different treatments. (d). Protein expression was tested by WB. ^*∗*^*P* < 0.05 vs. the control group. ^#^*P* < 0.05 vs. the erastin group. And *P* < 0.05 vs. the H/R group.

Firstly, we studied the effect of ATV on the ferroptosis of H9C2 cells.  Figure 1(a) showed that cell viability decreased in the erastin and H/R groups compared with the control group. Compared with the erastin or H/R groups, cell viability in the erastin + ATV and H/*R* + ATV groups increased (Figure 1(a)). Compared with the control group, cells in the erastin and H/R groups showed mitochondrial shrinkage, increased membrane density, and decreased mitochondrial crest number, which was characteristic of ferroptosis (Figure 1(b)). After ATV treatment, the volume of mitochondria and the number of mitochondrial cristae increased (Figure 1(b)). Next, we detected ROS in the cells. As shown in Figure 1(c), ATV reversed abnormally elevated ROS levels (DCFH-DA solution and C11-BODIPY fluorescent probe) in the erastin and H/R groups. GPX4 and SLC7A11 levels were lower in the erastin and H/R groups than in the control group, while ACSL4 and COX2 levels were higher in the erastin and H/R groups (Figure 1(d)). ATV treatment reversed this trend (Figure 1(d)). These results showed that ferroptosis in H9C2 cells was successfully induced by erastin and H/R and ATV significantly alleviated it.

### 3.2. ATV Regulated Cellular Iron Homeostasis of H9C2 Cells

**Figure 2:**
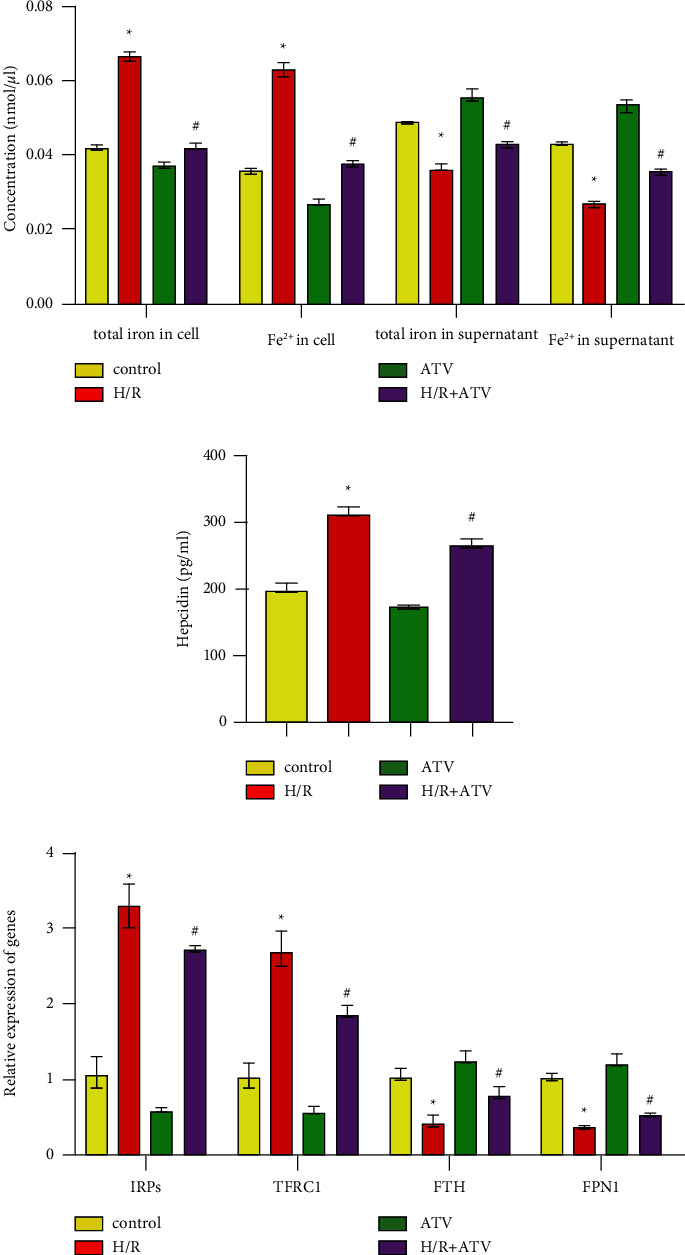
ATV regulated cellular iron homeostasis of H9C2 cells. (a–b). The concentration of total iron, Fe^2+^, and hepcidin was calculated by ELISA. (c). The relative expression of genes was measured by qPCR. ^*∗*^*P* < 0.05 vs. the control group. ^#^*P* < 0.05 vs. the H/R group.

We then investigated the effect of ATV on iron homeostasis in H9C2 cells. As shown in Figure 2(a). ATV treatment reduced abnormally elevated concentrations of total iron and Fe^2+^ in the cell of H/R group. At the same time, the concentration of total iron and Fe^2+^ in cell supernatant increased (Figure 2(a)). These results suggested that ATV could promote total iron and Fe^2+^ excretion from H9C2 cells. The expression levels of hepcidin, IRPs, and TFRC1 were significantly decreased, and the expression levels of FTH and FPN1 were increased in the H/*R* + ATV group than that in the H/R group (Figures 2(b) and 2(c)). All the results proved that ATV promoted iron ion excretion in H9C2 cells at the molecular level.

### 3.3. ATV Activated the SMAD7 Expression

**Figure 3:**
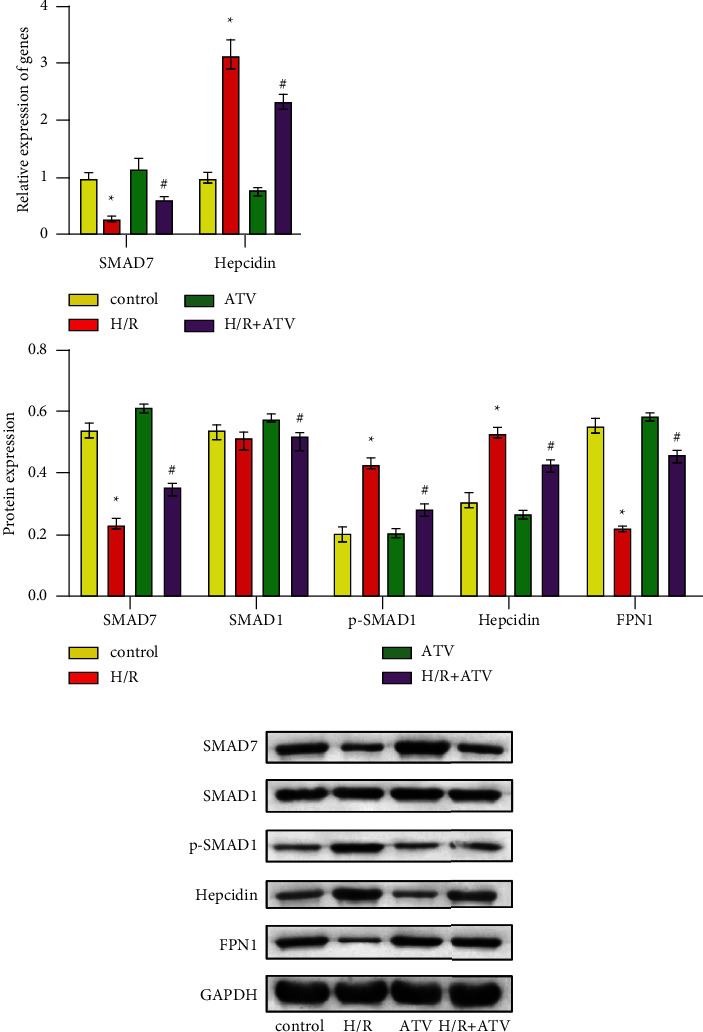
ATV activated the SMAD7 expression. (a). The relative expression of SMAD7 and hepcidin was detected by qPCR. (b). WB was used to analyze the proteins expression. ^*∗*^*P* < 0.05 vs. the control group. ^#^*P* < 0.05 vs. the H/R group.

To investigate the molecular mechanism of ATV in the H/R-induced H9C2 cells ferroptosis, we examined the expression of genes and proteins associated with the SMAD7 pathway. Compared with the control group, the expression of the SMAD7 gene in the H/R group decreased, while the expression level of hepcidin increased (Figure 3(a)). After treatment with ATV (the H/*R* + ATV group), the expression level of the SMAD7 gene increased, while the expression level of hepcidin decreased (Figure 3(a)). At the protein level, compared with the H/R group, the expression of SMAD7 and FPN1 in the H/*R* + ATV group increased, while the expression of p-SMAD1 and hepcidin decreased (Figure 3(b)). These results indicated that ATV could activate the expression of SMAD7 to regulate the hepcidin/FPN1 pathway.

### 3.4. ATV Inhibited Ferroptosis in H9C2 Cells through SMAD7

**Figure 4:**
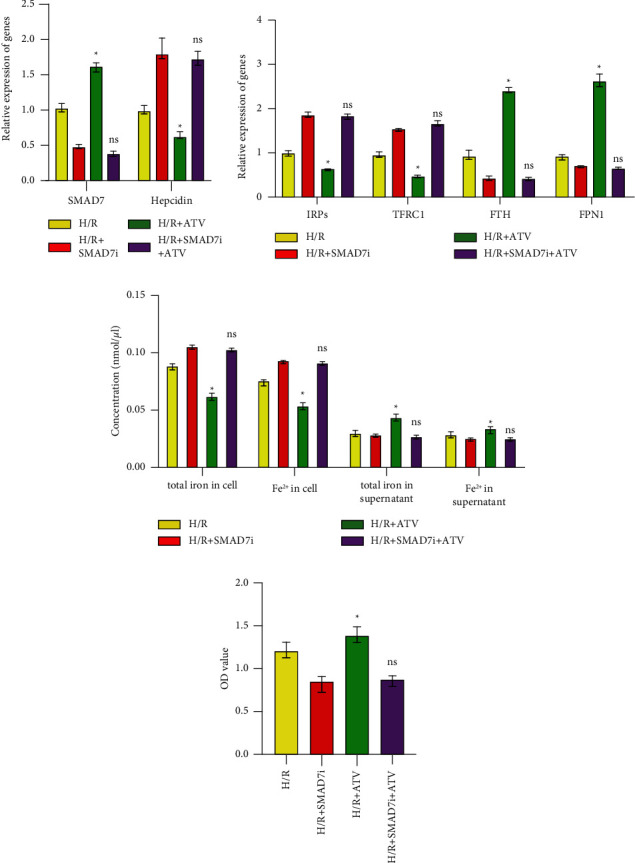
ATV inhibited ferroptosis in H9C2 cells through the SMAD7/hepcidin pathway. (a–b).The relative expression of SMAD7, hepcidin, IRPs, TFRC1, FTH, and FPN1 were detected by qPCR. (c). ELISA was utilized to determine the concentration of total iron and Fe^2+^. (d). The cell vitality was measured by CCK8. ^*∗*^*P* < 0.05 vs. the H/R group. ns: no significant difference vs. the H/*R* + SMAD7i group.

Then, we knocked down the SMAD7 gene in H9C2 cells. As shown in Figures 4(a) and 4(b), compared with the H/*R* + SMAD7i group, the gene expression levels of SMAD7, hepcidin, IRPs, TFRC1, FTH, and FPN1 in the H/*R* + SMAD7i + ATV group showed no significant difference.  Figure 4(c) showed that there was no significant difference in the total iron and Fe^2+^ between H/*R* + SMAD7i and H/*R* + SMAD7i + ATV groups. These results reflected that ATV treatment could not regulate iron homeostasis in H9C2 cells in the absence of SMAD7. CCK8 showed that there was no significant difference in cell viability between H/*R* + SMAD7i and H/*R* + SMAD7i + ATV groups (Figure 4(d)). These results demonstrated that the inhibition of ferroptosis in H9C2 cells by ATV was realized by regulating SMAD7.

### 3.5. ATV Resisted Rat Myocardial Ferroptosis by SMAD7/Hepcidin

**Figure 5:**
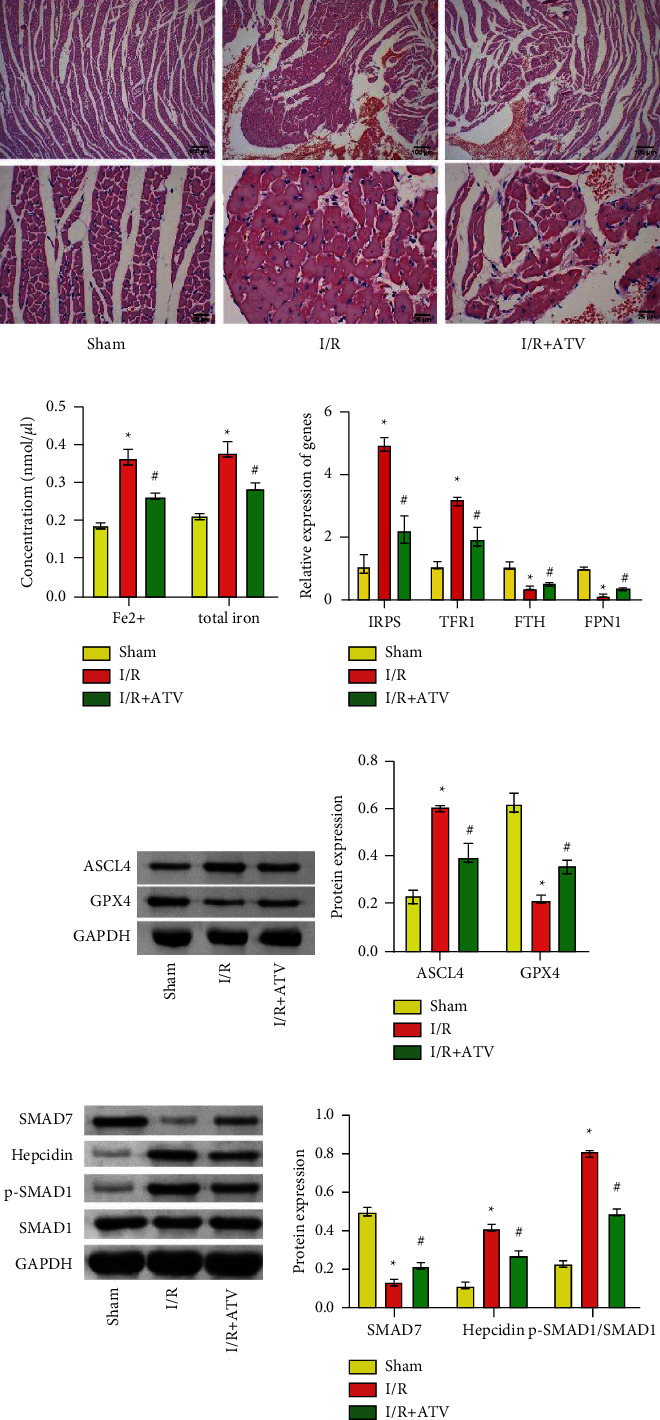
ATV improved ferroptosis in I/R rat myocardium by the SMAD7/hepcidin pathway. (a) Cardiac histopathology was observed by HE staining (100× and 400×). (b) The levels of Fe^2+^ and total iron were detected by ELISA. (c) The expression of IRPs, TFRC1, FTH, and FPN1 was detected by qPCR. (d–e) The expression of ACSL4, GPX4, SMAD7, SMAD1, p-SMAD1, and hepcidin proteins was detected by Western blot. ^*∗*^*P* < 0.05 vs. the sham group; ^#^*P* < 0.05 vs. the I/R group.

HE staining showed that myocardial tissue structure was intact and myocardial cells were arranged neatly in the Sham group (Figure 5(a)). In the I/R group, myocardial tissue cells were disordered, with swelling, necrosis, and myocardial boundary rupture (Figure 5(a)). The myocardial tissues of the I/*R* + ATV group were better than that of the I/R group (Figure 5(a)). Compared with the Sham group, the contents of Fe^2+^ and total iron in myocardial tissues of the I/R group were significantly increased (Figure 5(b)). ATV intervention down-regulated the levels of Fe^2+^ and total iron in I/R rat myocardial tissues (Figure 5(b)). In addition, the expressions of IRPs and TFR1 were increased, and the expressions of FTH and FPN1 were decreased in the I/R group (Figure 5(c)). ATV intervention inhibited the expression of IRPs and TFR1, and promoted the expression of FTH and FPN1 in I/R rat myocardium (Figure 5(c)). Protein expression analysis showed that compared with the Sham group, the expressions of ASCL4, hepcidin, and p-SMAD1 were increased, and the expressions of GPX4 and SMAD7 were decreased in myocardial tissues of the I/R group (Figures 5(d) and 5(e)). ATV intervention inhibited the expression of ASCL4, hepcidin, and p-SMAD1, and promoted the expression of GPX4 and SMAD7 in I/R rat myocardial tissues (Figures 5(d) and 5(e)).These results suggested that ATV improved ferroptosis in I/R rat myocardium by the SMAD7/hepcidin pathway.

## 4. Discussion

In general, increased iron uptake (e.g., due to TFR overexpression), reduced iron storage (e.g., due to ferritin knockdown or induction of ferritin phagocytosis), and impaired cellular iron output (e.g., due to FPN knockdown) enhanced the sensitivity of cells to ferroptosis [22].Although in many types of cancer, statins were thought to induce ferroptosis to exert antitumor effects [23]. But in other processes, ATV reduced epidermal growth factor-induced ROS production and up-regulated GPX4 protein expression in rat vascular smooth muscle [24]. Qing Li et al. were the first to demonstrate that fluvastatin protected endothelial cells from ox-LDL-induced ferroptosis and dysfunction [25]. In our study, ATV treatment mitigated erastin or H/R-induced ferroptosis in H9C2 cells. All these studies supported that ATV could block ferroptosis in H/R-induced H9C2 cells injury.

In sepsis-induced acute kidney injury mouse model, ATV (ATV/PTP TCeria NPs) has excellent antioxidant and antiapoptotic activities, which could protect mitochondrial structure and reduce renal injury [26].In addition, ATV attenuated isoflurane (1.5%) exposure-induced ROS generation and neuronal apoptosis in C57BL/6 mice [27]. ATV could also inhibit the generation of ROS in peripheral blood mononuclear cells of diabetic patients [28]. Our study found ATV reversed abnormally elevated ROS levels (DCFH-DA solution and C11-BODIPY fluorescent probe) in the erastin and H/R-induced H9C2 cells. All the studies proved ATV could inhibited ROS generation in H/R-induced H9C2 cells injury.

Ferroptosis was usually accompanied by substantial iron accumulation [29]. Transferrin receptor 1 (TFRC1) and FPN1 controlled iron inflow and outflow, respectively [30]. The stability of the former mRNA was positively regulated by iron regulatory proteins (IRPs), while the latter was negatively regulated by hepcidin [31]. Loss of FTH promoted cardiomyopathy through SLC7A11-mediated ferroptosis [30]. Our study found that ATV regulated intracellular iron homeostasis in erastin or H/R-induced H9C2 cells. All these studies supported that ATV could regulate intracellular iron homeostasis in H/R-induced H9C2 cells injury.

Previously, ATV intervention reduced SMAD7 protein expression in human atrial fibroblasts [32]. Statins inhibited angiotensin II-induced activation of SMAD and associated vascular fibrosis through a TGF-*β* independent process [33]. ATV promoted the antifibrotic effect on the myocardium by inhibiting SMAD2 activity [34]. Statins consume cholesterol to inhibit SMAD1 phosphorylation and nuclear translocation in cultured sensory neurons [35].Our study found that H/R-induced SMAD7 expression was up-regulated, and phosphorylation of SMAD1expression was decreased in H9C2 cells after ATV treatment. All these studies demonstrated the activation of SMAD7 by ATV in H/R-induced H9C2 cells and I/R rat myocardium.

In addition, we found that ATV intervention decreased the hepcidin expression and increased the FPN1 expression in H/R-induced H9C2 cells. In a study of patients with stage 3 and 4 chronic kidney disease, 6 months of ATV administration reduced hepcidin expression [6]. The BMP-SMAD pathway was a classic pathway for regulating iron by activating the IL-6/hepcidin signal and reducing serum iron and transferrin saturation [36]. Hepcidin was an important factor leading to iron overload in cerebral ischemia [37]. Knockdown of ferritin inhibited the increase of L-ferritin and decrease of FPN1 in I/R [38]. Our study proved ATV intervention up-regulated the expression of SMAD7 in H9C2 cells induced by H/R, thereby inhibiting the SMAD pathway and down-regulating the expression of hepcidin, and activating FPN1 to transport intracellular iron out of the cell.

ATV and its two active metabolites o-hydroxyatorvastatin (o-OH-ATV) and p-hydroxyatorvastatin (p-OH-ATV) are responsible for its HMG-CoA (3-hydroxy-3-methylglutaryl CoA) reductase inhibitory activity, while the corresponding inactive lactone form (LAC) was associated with myopathy [39].Our in vivo study firstly proved that ATV inhibited ferroptosis in I/R rat myocardium. However, its drug metabolism and stability in rats still need to be further evaluated. It was known that the porous polylactic acid membrane loaded with ATV hot gel has excellent anti-inflammation ability and good antiadhesion ability [40], which has great potential for clinical application of ATV in I/R myocardium.

H_2_O_2_ increased iron concentration in retinal pigment epithelial cells via the BMP6/SMAD/hepcidin axis, resulting in age-related macular degeneration [41].Inhibitory SMAD7 is a key repressor of hepcidin, the master regulator of systemic iron homeostasis [42]. ATV therapy improves isoprenaline attack-induced cardiac function and remodeling by reducing ferroptosis [43]. ATV reverses barrier dysfunction, oxidative stress, and ferroptosis in human lung microvascular endothelial cells induced by cigarette smoke extract [44].Our *in vivo* and *in vitro* study proved that ATV inhibited ferroptosis in I/R rat myocardium through the SMAD7/hepcidin pathway, which provides new insights for the clinical application of ATV in I/R myocardium.

## 5. Conclusion

ATV intervention blocked erastin or H/R-induced ferroptosis in H9C2 cells by activating SMAD7 expression and thereby down-regulating the hepcidin/FPN1 pathway. The *in vivo* study also demonstrated that ATV inhibited ferroptosis in I/R rat myocardium through the SMAD7/hepcidin pathway.

## Figures and Tables

**Table 1:** qPCR primer sequences.

Gene	Sequences (5′-3′)	Product length (bp)
TFR1	F: GCCACAAGCCAAACAATATCCG	250
	R: ACACTGCTCCCGATAATGTGA	

IRP1	F: TATGCGTGGAACCCCAAGTC	157
	R: TGTTCCCCGCTGGAGAGATA	

TFH	F: TGAGCCCTTTGCAACTTCGT	217
	R: CTTCAGGGCCACATCATCCC	

FPN1	F: CAGGCTTAGGGTCTACTGCG	210
	R: GAGGAACCGGAGATAGCACG	

SMAD7	F: GAGTCTCGGAGGAAGAGGCT	84
	R: CTGCTCGCATAAGCTGCTGG	

Hamp	F: CAGACGAGACAGACTACGGC	95
	R: TCTCGCTTCCTTCGCTTCAG	

GAPDH	F: ACAGCAACAGGGTGGTGGAC	252
	R: TTTGAGGGTGCAGCGAACTT	

## Data Availability

The data used to support the findings of this study are available from the corresponding author upon request.
